# Supplementary material for: Impact of overdose prevention sites during a public health emergency in Victoria, Canada
Source: PLoS One. 2020 May 21;15(5):e0229208. doi: 10.1371/journal.pone.0229208 (PMC7242015; doi:10.1371/journal.pone.0229208)
Supplement: S1 Data — (DOCX) [file pone.0229208.s001.docx]

**Interview Guide – Service Users**

Semi-structured Questions

1. What prompted you to use the overdose prevention site?
2. When did you start using the site?
3. How often do you use the site? (Be specific as to number of times daily, weekly, monthly)
4. How would you describe this site to someone who hasn’t used one?
5. What are your expectations of this service
6. How well does this site meet your needs/expectations
7. Tell me a bit about a typical visit to the site? What happens first, second? What is the general feeling (vibe) at the site?
8. Tell me about some of the policies at the site? What do you think of these policies?
9. What has been a positive experience at the site?
10. What has been a negative experience?
11. What are your thoughts on how the site is set up?
    1. What do you like about the site? What do you not like?
    2. What is the role of staff at the site?
    3. What are your relationships like with the staff? Distinguish between peers and other staff if possible?
12. What would you describe as working well that should not be changed?
13. What would you say should be changed to improve the site?
14. Have you used other overdose prevention sites?
15. If you have used other sites, how does this site compare to other overdose prevention sites?
16. How do does this site compare to a **supervised consumption service**? What do you think would be different?
17. What difference (impact) has this site had on
    1. Your health (e.g physical, mental, emotional, spiritual)
    2. Your drug use
    3. Stigma or feelings of safety
    4. Access to other services (e.g referrals to housing, social services etc)
    5. Relationships with the organization
    6. Relationship with other people at the site
18. What, if anything, facilitates you to use the site? What acts as barriers?
19. What are the benefits of having the site? What are the risks in using the site?
20. How well accepted do you think the site is within the organization? Within the city? How has having this site changed the organization? The city?
21. Besides the overdose prevention sites, where else do you usually use drugs?
    1. What is the reason you use these other places?
22. What do you think it is important for us to learn from this overdose emergency?
23. Moving forward, what do you recommend?

**Demographics – Service Users – Interviewer verbally asks participants these questions**

**1. What type of accommodation do you currently live in?**

- Owned house/apartment (alone or shared)
- Rented house/apartment (alone or shared)
- Foster home
- Boarding house / hostel / single room occupancy hotel (SRO)
- Student residence
- Shelter / refuge
- Drug Treatment Residence
- Squat
- No fixed address/couch surfing/staying with friends
- Other (specify) ______________________

**2. Is your current housing situation stable? (e.g. Will you be able to stay as long as you want to)**

- ¨Yes
- No
- N/A or No fixed address
- Don’t know
- Refused

1. **What year were you born? [If unsure, ask for best estimate]**

| - ____/_______/______   DAY/MONTH/YEAR | - DON’T KNOW | - DECLINE TO ANSWER |
| --- | --- | --- |

1. **What gender do you identify with? [DO NOT READ CATEGORIES]**

| - MALE - FEMALE | - TRANSGENDER - OTHER RESPONSE__________________ | - DON’T KNOW - DECLINE TO ANSWER |
| --- | --- | --- |

**5. If other response is selected, please specify:**

| - TRANS WOMAN - TRANS MAN - TWO-SPIRIT | - GENDERQUEER - GENDERFLUID - ANDROGYNOUS | - NON-BINARY - INTERSEX - NONE OF THE ABOVE |
| --- | --- | --- |

1. **What ethnic group or family background do you identify yourself as? (CHECK ALL THAT APPLY)**

- White
- Chinese
- South Asian (East Indian, Pakistani, Sri Lankan etc)
- Black
- Fillipino
- Latin American
- Southeast Asian (E.g. Cambodian, Indonesian, Laotian, Vietnamese etc) Arab (e.g Arb speaking Maghrebi)
- West Asian (e.g. Afhan, Iranian, Israeli, Turk, etc)
- Japanese
- Korean
- Indigenous If yes, do you identify as First Nations, Inuit, Metis)
- Other (Please Specify) ________________________________
- Don’t know
- Refused

**7. Throughout your life, how long have you been living in Greater Victoria?**

**[Check one. Note: If person has been living in Greater Victoria periodically**

**throughout their life, mark cumulatively over these periods]**

| - LESS THAN A MONTH - 1 MONTH TO LESS THAN 1 YEAR - 1 year to less 5 years - 5 years to less than 10 years - 10 years or more - Whole life - Don’t know - Decline to Answer |
| --- |

**8. If you moved to Greater Victoria in the last year, where were you living prior to coming here?**

| - COMMUNITY NAME____________________ AND PROVINCE____________________ - OR COUNTRY - DON’T KNOW - DECLINE TO ANSWER |
| --- |

**9. What is the highest level of education you have completed? (check ONE box only)**

- No schooling
- Some elementary schooling
- Completed elementary school
- Some high school
- Completed high school
- Some community college
- Some technical school
- Completed community college
- Completed technical school
- Some university
- Completed Bachelor’s Degree
- Post graduate training: MA, MSc., MSW
- Post graduate training: PhD, “Doctorate”
- Professional degree (Law, Medicine, Dentistry)
- Don’t know
- Refused

1. **Where do you get your money from?** [May provide examples. Select all that apply]

| - EMPLOYMENT - INFORMAL/SELF-EMPLOYMENT (E.G., BOTTLE RETURNS, PANHANDLING) - EMPLOYMENT INSURANCE - WELFARE/INCOME ASSISTANCE - DISABILITY BENEFIT | - SENIORS BENEFITS (E.G., CPP/OAS/GIS) - CHILD AND FAMILY TAX BENEFITS - MONEY FROM FAMILY/FRIENDS - OTHER SOURCE _______________________ - NO INCOME - DECLINE TO ANSWER |
| --- | --- |

| Y=Yes  N=No  DK=Don’t know  RF=Refused | **During the past 12 months have you used any…** | **Has your substance use changed since using the site?**  **🡹 = increased**  **🡻= decreased**  **same = stayed the same** | **Number of days used in the**  **past 30 days**  Write (0-30) | **How have you used in the past 30 days? (Circle all that Apply)** |
| --- | --- | --- | --- | --- |
| Alcohol? | Y N RF DK | 🡹 🡻 same RF | ..............Days |  |
| Codeine products like Tylenol #3, Tylenol #1, 292's or 222's? **T3’s** | Y N RF DK | 🡹 🡻 same RF | ..............Days | Smoke Inject Orally Other |
| Oxycodone products such as Percocet or Percodan? **Perc’s, Oxies** | Y N RF DK  If yes, indicate   - Prescribed - Not Prescribed | 🡹 🡻 same RF | ..............Days | Smoke Inject Orally Other |
| Other Opioid products such as hydromorphone, Dilaudid, Hydromorph Contin, morphine, MS Contin, or Demerol?  **Dilly’s, Dilly 8** | Y N RF DK  If yes, indicate   - Prescribed - Not Prescribed | 🡹 🡻 same RF | ..............Days | Smoke Inject Orally Other |
| Stimulants?  (i.e. Ritalin, Concerta, Adderall, Dexedrine) **Uppers, Dex** | Y N RF DK  If yes, indicate   - Prescribed - Not Prescribed | 🡹 🡻 same RF | ..............Days | Smoke Inject Orally Other |
| Sedatives?  (i.e. diazepam, Valium, lorazepam, Ativan, alprazolam, Xanax, clonazepam, Rivotril) **Benzo’s** | Y N RF DK  If yes, indicate   - Prescribed - Not Prescribed | 🡹 🡻 same RF | ..............Days | Smoke Inject Orally Other |
| Marijuana?  (i.e. marijuana, hashish, hash oil or other cannabis derivatives) **Grass, Weed** | Y N RF DK  If yes, indicate   - Prescribed - Not Prescribed | 🡹 🡻 same RF | ..............Days | Smoke Inject Orally Other |
| Methadone? | Y N RF DK  If yes, indicate   - Prescribed - Not Prescribed | 🡹 🡻 same RF | ..............Days | Smoke Inject Orally Other |
| **Fentanyl?**  (Knowingly or unknowingly) | Y N RF DK | 🡹 🡻 same RF | ..............Days | Smoke Inject Orally Other |

| Cocaine or Crack? **Rock** | Y N RF DK | 🡹 🡻 same RF | ..............Days | Smoke Inject Orally Other |
| --- | --- | --- | --- | --- |
| Speed or Methamphetamines? **Side, , Jib, Ice** | Y N RF DK | 🡹 🡻 same RF | ..............Days | Smoke Inject Orally Other |
| Ecstasy? **E, M, MDMA, MDA, MDEA** | Y N RF DK | 🡹 🡻 same RF | ..............Days | Smoke Inject Orally Other |
| Hallucinogens?  (i.e. PCP, LSD, acid, magic mushrooms, mescaline or angel dust?) **Mesc, shrooms** | Y N RF DK | 🡹 🡻 same RF | ..............Days | Smoke Inject Orally Other |
| Heroin? **Down, Pants, Smac** | Y N RF DK | 🡹 🡻 same RF | ..............Days | Smoke Inject Orally Other |
| Salvia? | Y N RF DK | 🡹 🡻 same RF | ..............Days | Smoke Inject Orally Other |

| Y=Yes  N=No  DK=Don’t know  RF=Refused | **During the past 12 months have you…** | **Has your substance use changed since using the site?** | **Number of days used in the**  **past 30 days**  Write (0-30) |
| --- | --- | --- | --- |
| Drank non-beverage alcohol such as rubbing alcohol, hand sanitizer, etc? | Y N RF DK | 🡹 🡻 same RF | ..............Days |
| Sniffed glue, gasoline, other solvents? (Inhalents) | Y N RF DK | 🡹 🡻 same RF | ..............Days |
