# Supplementary material for: Impact of overdose prevention sites during a public health emergency in Victoria, Canada
Source: PLoS One. 2020 May 21;15(5):e0229208. doi: 10.1371/journal.pone.0229208 (PMC7242015; doi:10.1371/journal.pone.0229208)
Supplement: S2 Data — (DOCX) [file pone.0229208.s002.docx]

**Interview Guide – Staff**

**Semi-structured Questions** – *Note: Questions about implementation may only be relevant to those staff that were involved in establishing the service rather than service provision.*

1. Please describe to me your role at the site?
   1. What is your position/title?
   2. How long have you worked at the site?
2. How did the site initially get started here and how did you and others react to the idea of setting up this service here?
3. Did you see a need for this site here? Why or Why not?
4. What services were you already offering to people who use drugs, before the site was set-up?
   1. How were people being treated?
   2. Do you perceive the site being a better alternative or not? Why or why not?
5. Can you tell me about the experience of getting this site implemented, how difficult was it to implement this site in this facility?
   1. What were the barriers? What were some facilitators?
   2. Are there ways in which this site has changed the agency here or the city in general?
6. Can you describe this site and the services to me?
   1. Can you describe the physical space/configuration and location in the facility?
   2. What is the general feeling (vibe) at the site?
   3. What are the different staff positions/roles?
   4. Tell me about the policies at the site? What do you think of these policies?
   5. How is the service funded?
   6. What are the goals of this service?
   7. How is this site similar or different than other overdose prevention sites?
7. Tell me about a typical day at the site?
8. Why do people want to use this service?
   1. What do people who use the service think of it?
   2. Does it meet their needs and expectations?
9. Do you think the service works better for some people than others? Who, what circumstances?
10. Overall, how successful do you think the site is? *(note: however the person defines success)*
    1. What would you describe as working well that should not be changed?
    2. What would you say should be changed to improve the site?
11. How do you define harm reduction? How does this site fit with that definition?
    1. Have you noticed any notable successes or challenges in implementing harm reduction principles in this site?
    2. What is your experience in harm reduction?
12. How is this site similar or different than a **supervised consumption service**? What do you think would be different?
13. What is the most important thing you have learned from working at the site?
14. What difference has the site made for yourself, your agency, other agencies, the people using the site?
15. Moving forward, what do you recommend?
    1. Would you recommend continuing the site here? Why or why not?
    2. Would you recommend these services in other locations? Why or why not?
    3. Do you have any suggestions for other places that have not yet stated these services?
    4. How would you improve this service/site?
16. What do you think we need to learn from this overdose crisis?

**Demographics – Interviewer verbally asks participants these questions.**

1. **What year were you born? [If unsure, ask for best estimate]**

| - ____/_______/______   DAY/MONTH/YEAR | - DON’T KNOW | - DECLINE TO ANSWER |
| --- | --- | --- |

1. **What gender do you identify with? [DO NOT READ CATEGORIES]**

| - MALE - FEMALE | - TRANSGENDER - OTHER RESPONSE__________________ | - DON’T KNOW - DECLINE TO ANSWER |
| --- | --- | --- |

**3. If other response is selected, please specify:**

| - TRANS WOMAN - TRANS MAN - TWO-SPIRIT | - GENDERQUEER - GENDERFLUID - ANDROGYNOUS | - NON-BINARY - INTERSEX - NONE OF THE ABOVE |
| --- | --- | --- |

1. **What ethnic group or family background do you identify yourself as? (CHECK ALL THAT APPLY)**

- White
- Chinese
- South Asian (East Indian, Pakistani, Sri Lankan etc)
- Black
- Fillipino
- Latin American
- Southeast Asian (E.g. Cambodian, Indonesian, Laotian, Vietnamese etc) Arab (e.g Arb speaking Maghrebi)
- West Asian (e.g. Afhan, Iranian, Israeli, Turk, etc)
- Japanese
- Korean
- Indigenous If yes, do you identify as First Nations, Inuit, Metis)
- Other (Please Specify) ________________________________
- Don’t know
- Refused

**5. What is the highest level of education you have completed? (check ONE box only)**

- No schooling
- Some elementary schooling
- Completed elementary school
- Some high school
- Completed high school
- Some community college
- Some technical school
- Completed community college
- Completed technical school
- Some university
- Completed Bachelor’s Degree
- Post graduate training: MA, MSc., MSW
- Post graduate training: PhD, “Doctorate”
- Professional degree (Law, Medicine, Dentistry)
- Don’t know
- Refused
